# Knowledge of obstetric danger signs and associated factors among pregnant women attending antenatal care services at Thai community hospital

**DOI:** 10.12688/f1000research.131267.1

**Published:** 2023-07-19

**Authors:** Pruk Koovimon, Kasiphak Kaikaew, Khanittha Mahoree, Thanapob Bumphenkiatikul

**Affiliations:** 1 Wang Saphung Hospital, Wang Saphung, Loei, 42130, Thailand; 2 Department of Physiology, Faculty of Medicine, Chulalongkorn University, Pathumwan, Bangkok, 10330, Thailand; 3 Center of Excellence in Transgender Health (CETH), Faculty of Medicine, Chulalongkorn University, Pathumwan, Bangkok, 10330, Thailand; 4 Division of Academic Affairs, Faculty of Medicine, Chulalongkorn University, Pathumwan, Bangkok, 10330, Thailand

**Keywords:** Awareness, Women's health, Complications, Maternal mortality, Delivery, Healthcare, Health education, Postpartum

## Abstract

Background: To decrease preventable maternal mortality, providing health education to all parties is mandatory. Good knowledge, including awareness of pregnant women regarding obstetric danger signs (ODS), leads to appropriate practices and services. The knowledge of ODS varies among countries and regions. Since the data in rural regions of Thailand remains unavailable, this study aimed to identify the prevalence of good ODS knowledge and associated factors among pregnant women attending antenatal services at a Thai community hospital.

Methods: We performed a cross-sectional, analytical study in 415 singleton pregnant women who visited the antenatal clinic at Wang Saphung Hospital, Loei, Thailand. A well-trained research assistant interviewed all participants using the data record form containing twenty items on the demographic and obstetric data and sixteen items on ODS knowledge. An ODS score of at least 75% (12 points) was considered a good level of knowledge.

Results: A total of 275 participants (66.27%) had good knowledge of ODS. The most recognized ODS was vaginal bleeding whereas the least recognized ODS during pregnancy was convulsion; the least recognized ODS during labor and delivery was retained placenta. Multivariate regression analysis showed that the predictive factors of good OBS knowledge included a higher education level, maternal age of at least 20 years, and having medical personnel as a source of knowledge.

Conclusions: In a rural setting of Thailand, two-thirds of pregnant women had good ODS knowledge. Identifying those at risk for fair and poor ODS knowledge and prompt management for the vulnerable subgroups might help decrease maternal mortality.

## Introduction

The maternal mortality ratio (MMR) is one of the indicators of the public health status of a specific region. Several global authorities devised a long-term plan to reduce global MMR. In 2000, the United Nations (UN) declared the Millennium Development Goals, which included “improving maternal health,” as Millennium Development Goal 5 (MDG-5). The MDG-5 aimed for a 75% reduction in the global MMR. ^1^ In 2015, the United Nations Department of Economic and Social Affairs (UNDESA) and all member countries agreed to aim to accomplish the Sustainable Development Goals (SDG) by 2030. The target of SDG Goal 3 is to “ensure healthy lives and promote well-being for all at all ages.” This goal includes “reducing the global MMR to less than 70 per 100,000 births, with no country having a maternal mortality rate of more than twice the global average.” ^2^ Having these goals consistently included in international plans implicitly reflects the global significance of this unresolved situation.

Globally, the MMR is decreasing. ^3^ The index differs among countries, ranging from 23.8 deaths per 100,000 100,000 live births in the US to 442 deaths per 100,000 births in Africa. ^4^^,^^5^ Thailand’s maternal mortality rate (MMR) was 48.0 deaths per 100,000 live births in 2008. ^6^ It fell to 37.0 deaths per 100,000 live births in 2017. ^7^ Though the low baseline ratio met SDG Goal 3, achieving “equity in MMR for vulnerable populations at the sub-national level” is still the country’s target. ^8^

To decrease preventable maternal mortality, providing health education to all parties involved in healthcare, including patients, families, communities, and medical personnel, is mandatory. Good knowledge will lead to appropriate attitudes and practices, i.e., prompt referral to appropriate medical services, and thus leading to a decrease in preventable maternal mortality. Unawareness of obstetric danger signs (ODS) will delay the decision to seek proper care and eventually lead to morbidity and mortality. ^9^

The knowledge of ODS varies among countries and regions. The prevalence of mothers with good knowledge of ODS seems to be lower in some countries or in some rural areas of the countries that have a higher MMR. Several studies on ODS knowledge have been conducted in developing countries, focusing on urban and rural areas. Most of the studies found that less than half of the study population had good knowledge of ODS. In addition, the prevalence of good knowledge or awareness of ODS in rural areas was lower than in urban areas. One study was conducted to examine the prevalence of mothers with good knowledge of ODS and associated factors in a tertiary care university hospital in the capital city of Thailand and found that the prevalence of mothers with the good knowledge was around 60%. ^10^ Currently, no data is available regarding the prevalence of mothers with good knowledge of ODS and associated factors in rural areas of Thailand, which are the subnational region at risk for higher MMR. Thus, our study aimed to identify the prevalence of mothers with good knowledge of ODS and associated factors among pregnant women attending an antenatal care clinic at a community hospital in a Northeastern province of Thailand. In addition, we also aimed to identify the variables that could predict the ODS knowledge of the pregnant women.

## Methods

### Ethics

The study protocol was approved by the Human Research Ethics Committee, Loei Provincial Public Health Office, (approval number: 0032.009/5503). Data collection was allowed by the director of Wang Saphung Hospital. Before the data collection, we obtained written informed consent from the participants or parents for participants under the legal age of consent.

### Study design and participants

This study is a cross-sectional, analytical study and was reported according to the STROBE statement for cohort studies. Participants were women with an ultrasound-confirmed singleton pregnancy who could understand Thai and had their antenatal clinic visit(s) at Wang Saphung Hospital, Loei Province, from 1 ^st^ July 2021 to 30 ^th^ September 2022. Those who were medical personnel or had any prior antenatal clinic visits in any other healthcare setting were excluded from the study to avoid contamination of ODS education during current gestation before participation. The study gave no incentives to participants. Participants’ partners were not included into this study.

### Data collection

All participants were informed about the study’s purposes, procedures, risks, and benefits. They were ensured that the decision was entirely voluntary. They could refuse or withdraw from the study at any time. Refusal, withdrawal, or having poor ODS knowledge would not affect the benefits or quality of care provided. A well-trained research assistant interviewed all participants.

The process took place in a private room during the clinic’s waiting period so as not to interfere with the care provided. The interview took approximately 30 minutes to complete. The research assistant completed two data record form sections. Section 1 consisted of twenty items on the demographic and obstetric data, which included parity, number of antenatal visits, and gestational age at the interview. Section 2 consisted of sixteen pre-coded closed-ended items on knowledge of ODS: twelve of which were knowledge during pregnancy and four were knowledge during labor and delivery. Participants were asked to spontaneously list all signs they perceived as dangerous or would urge them to seek proper care. The research assistant checked all ODS mentioned by each participant, then started asking for the participant’s knowledge of each of the rest of the ODS in section 2 of the data record form. Each response that acknowledged each ODS as dangerous was given one point. Zero point was given for unawareness of each ODS. We considered a score of at least 75% (12 points) to be a good level of knowledge, 50–74% (6–11 points) to be a fair level of knowledge, and 0–49% (0–5 points) to be a poor level of knowledge. The data collection process was derived from studies conducted in Thailand and Malaysia. ^10^^,^^11^ The Cronbach‘s alpha for ODS items was equaled to 0.89, previously mentioned in one study with comparable participants, which indicated a good reliability of the record form. ^11^

### Sample size justification

We calculated the sample size based on a previous study with a similar research design conducted by Kaewkiattikun *et al*., ^10^ in a medical school in an urban area of Thailand. We applied the power of 80% and a confidence level of 95% to determine the difference between groups. After adding an additional 10% to account for missing data, a total of 415 participants were recruited for this study. The study used a simple random sampling method. We limited the number of participants per day to 10 to ensure the quality of care and the data collected.

### Data analysis

To analyze the data, a statistician used SPSS version 29 (IBM, Armonk, NY, USA). For the analysis of categorical data between groups, the Chi-square test was employed. Multivariate logistic regression analysis was used to identify independent variables with good knowledge of ODS. The results were presented in odds ratios and 95% confidence intervals (CI). P-values less than 0.05 were regarded as statistically significant.

## Results

There were 415 eligible pregnant women at the end of the enrolment. Of all these participants, 275 (66.27%) had good knowledge of ODS, 101 (24.34%) had fair knowledge, and 39 (9.40%) had poor knowledge. The most recognized ODS was vaginal bleeding, which accounted for 92.29% of the reported ODS during pregnancy and 80.96% of the reported ODS during labor and delivery. The least recognized ODS during pregnancy was convulsion (68.19%), while the least recognized ODS during labor and delivery was retained placenta (63.61%). The detailed results of the knowledge of ODS among antenatal women are shown in Table 1.

**Table 1.** The knowledge of ODS among antenatal women.

Variables	Number (N=415)	Percentage
Knowledge of ODS		
Good (≥75%, ≥12 points)	275	66.27
Fair (50–74%, 6–11 points)	101	24.34
Poor (0–49%, 0–5 points)	39	9.40
ODS		
During pregnancy		
1. Vaginal bleeding	383	92.29
2. Decreased fetal movement	377	90.84
3. Uterine contraction	354	85.30
4. Severe nausea and vomiting	344	82.89
5. Epigastric pain	284	68.43
6. Severe abdominal pain	345	83.13
7. Severe headache	299	72.05
8. Shortness of breath	335	80.72
9. Fluid flowing from the vagina	344	82.89
10. Swelling body	305	73.49
11. Blurred vision	285	68.67
12. Convulsion	283	68.19
During labor and delivery		
1. Vaginal bleeding	336	80.96
2. Prolonged labor	302	72.77
3. Convulsion	279	67.23
4. Retained placenta	264	63.61

ODS refers to obstetric danger signs.

We classified those who knew at least 12 items out of 16 items (75%) as having a good level of ODS knowledge, 6–11 items (50–74%), and 0–5 items (0–49%) as having a fair level and a poor level of ODS knowledge, respectively. Using the Chi-square test, the identified factors that were significantly between the good and the fair/poor knowledge groups included participants’ age, education, occupation, marital status, gravida, and source of the ODS knowledge. After using the multivariate regression analysis to identify which of these characteristics were statistically significant predictors of good ODS knowledge, we found that participants’ age, education, and source of the ODS knowledge were predictors of good ODS knowledge, whereas participants’ occupation, marital status, and gravida were not statistically significant predictors of good ODS knowledge. The detailed results of the demographic characteristics of participants and their association with the level of ODS knowledge are shown in Table 2.

**Table 2.** Demographic characteristics of participants and association with the level of ODS knowledge.

Characteristic	Number (N=415)	Level of knowledge		*Chi p*-value	Multivariate regression		
		Good	Fair/Poor		AOR	95% CI	*p*-value
Age (year)				<0.001			
Below 20	36 (8.67)	13 (3.13)	23 (5.54)		1.000	Ref	Ref
20-35	319 (76.87)	218 (52.53)	101 (24.34)		3.064	1.431-6.562	0.004
Above 35	60 (14.46)	44 (10.6)	161 (38.8)		2.729	1.040-7.159	0.041
Participant's education				0.044			
High school or lower	339 (81.69)	217 (52.29)	122 (29.40)		1.000	Ref	Ref
Bachelor's degree or higher	76 (18.31)	58 (13.98)	18 (4.34)		2.586	1.353-4.942	0.004
Participant's occupation				0.009			
Employed	146 (35.18)	101 (24.34)	45 (10.84)		1.000	Ref	Ref
Farming and agriculture	70 (16.87)	42 (10.12)	28 (6.75)		0.854	0.450-1.621	0.630
Trade and commerce	125 (30.12)	93 (22.41)	32 (7.71)		1.379	0.787-2.414	0.261
Unemployed/Student	74 (17.83)	39 (9.4)	35 (8.43)		0.660	0.353-1.235	0.194
Monthly income (THB)				0.590			
Below 10,000	159 (38.31)	104 (25.06)	55 (13.25)		NA	NA	NA
10,001-20,000	170 (40.96)	109 (26.27)	61 (14.7)		NA	NA	NA
20,001-30,000	65 (15.66)	46 (11.08)	19 (4.58)		NA	NA	NA
Above 30,000	21 (5.06)	16 (3.86)	5 (1.2)		NA	NA	NA
Marital status				0.023			
Separated/divorced	55 (13.25)	29 (6.99)	26 (6.27)		1.000	Ref	Ref
Married	360 (86.75)	246 (59.28)	114 (27.47)		1.798	0.964-3.352	0.065
Gravida				0.006			
Primigravida	166 (40)	97 (23.37)	69 (16.63)		1.000	Ref	Ref
Multigravida	249 (60)	178 (42.89)	71 (17.11)		1.525	0.959-2.425	0.075
Current gestational age				0.606			
Below 20 weeks	326 (78.55)	213 (51.33)	113 (27.23)		NA	NA	NA
21 weeks or above	88 (21.2)	61 (14.7)	27 (6.51)		NA	NA	NA
Gestational age at first ANC visit				0.205			
Below 12 weeks	234 (56.39)	149 (35.9)	85 (20.48)		NA	NA	NA
12 weeks or above	181 (43.61)	126 (30.36)	553 (133.25)		NA	NA	NA
Number of ANC visits				0.290			
<4	237 (57.11)	152 (36.63)	85 (20.48)		NA	NA	NA
≥4	178 (42.89)	123 (29.64)	55 (13.25)		NA	NA	NA
Source of ODS knowledge				0.007			
Medical personnel	289 (69.64)	205 (49.4)	84 (20.24)		2.549	1.455-4.466	0.001
Family members	40 (9.64)	24 (5.78)	16 (3.86)		1.778	0.773-4.090	0.176
Friends/Media/Other	86 (20.72)	46 (11.08)	40 (9.64)		1.000	Ref	Ref
Source of help when encountering ODS				0.297			
Medical personnel	340 (81.93)	231 (55.66)	109 (26.27)		NA	NA	NA
Family members	57 (13.73)	33 (7.95)	24 (5.78)		NA	NA	NA
Friends/Other	18 (4.34)	11 (2.65)	7 (1.69)		NA	NA	NA

ODS, obstetric danger signs; AOR, adjusted odd ratio; CI, confident interval; THB, Thai Baht; ANC, Antenatal care; Ref, Reference; NA, not analyzed with logistic regression since the *Chi-square p*-value of the variables was greater than 0.05.

## Discussion

In a community hospital-based antenatal care services in a Northeastern province of Thailand, we found that about two-thirds of the pregnant women had a good level of knowledge regarding ODS. We also found that the factors that were associated with the good ODS knowledge included age, education level, and source of the ODS knowledge that the pregnant women obtained the information.

The prevalence of good ODS knowledge in our study is higher than those in previous studies. One study in a Thai university hospital in an urban area reported a prevalence of 59.8% ^10^ and another study in a teaching and referral hospital in Malaysia reported a prevalence of 48.3%. ^11^ The finding that the prevalence of good ODS knowledge in Thai pregnant women was slightly higher than that of the Malaysian study might be due to the different score cut-off levels of good knowledge of ODS, that is, 80% (16 out of 20 items) in the Malaysian study whereas 75% (12 out of 16 items) in our study and the other Thai study. Several studies reported the knowledge of ODS among pregnant women in many countries, including India, ^12^^,^^13^ Nepal, ^14^^,^^15^ Malaysia, ^11^ Ethiopia, ^16^^–^^28^ Nigeria, ^29^ Tanzania, ^30^ Egypt, ^31^ Jordan, ^32^ Congo, ^33^ and Uganda. ^34^ The fact that the prevalence of mothers with good knowledge of ODS differed among studies could be because of the difference in participants’ demographic characteristics and the definition of good knowledge of ODS in each study. Overall, the prevalence of mothers with good knowledge of ODS was the lowest in Africa, ^16^^–^^29^^,^^35^^,^^36^ especially in the remote area where most participants received lower education.

For the knowledge of each ODS among the study population, we found that vaginal bleeding is the most mentioned ODS during pregnancy and during labor/delivery among participants. Less than 70% of the participants reported epigastric pain, blurred vision, and convulsion during pregnancy as an ODS. A similar proportion mentioned convulsion and retained placenta during labor/delivery as ODS. These findings were congruent with the previous study in Thailand. ^10^ Since vaginal bleeding is a visible, genital organ–related sign, it is easily recognized as an ODS among pregnant women. In contrast, since epigastric pain, blurred vision, and convulsion are symptoms of other organ systems, it was more complicated to educate pregnant women that these symptoms must also be perceived as an ODS.

For the secondary objective of the study, the predictive factors of good knowledge of ODS, we found that higher maternal education of at least a Bachelor’s degree, compared to high school or lower education, is one of the significant predictors. This finding is similar to the study from the Thai university hospital. ^10^ Studies in other Asian countries, including Malaysia ^11^ and Jordan, ^32^ also found that higher maternal education was a significant predictive factor of good knowledge of ODS. Several studies from the African region, ^29^^,^^37^ especially Ethiopia, ^20^^,^^21^^,^^24^^–^^26^^,^^28^^,^^38^ where women received lower education, emphasized the predictive value of women’s education on the level of ODS knowledge. The level of education could be the source of the quality of ODS knowledge. Women with a lower education level could have more difficulty in understanding healthcare information about the importance of ODS given by others. The lower level of education might reflect lower opportunities for women since they might share poor attitudes and misinformation with peers at the same level of education. A study in Africa reported that ODS was perceived as a natural process of pregnancy or related to witchcraft. ^39^ This attitude was a significant barrier, preventing the women facing ODS from seeking proper help.

Another predictive factor of the better knowledge of ODS in our study was maternal age. Age was a good predictive factor in studies from Malaysia, ^11^ Ethiopia, ^20^^,^^21^^,^^24^^,^^27^^,^^28^^,^^38^ Tanzania, ^30^ Nigeria, ^29^ South Africa, ^35^ and Zambia. ^37^ Women with more advanced ages had better knowledge of ODS. This may be attributed to several hypotheses. Having better ODS knowledge is an essential indicator of pregnancy preparedness. Teenage pregnancy is more unprepared than pregnancy in adulthood. ^40^ Being an adult also means having more mature neurological development, awareness, and experience than being teenagers. Having more experience in older pregnant women leads to gaining both personal experience and information from experience of other people during their pregnancies. This hypothesis could explain our finding that women with multigravida tended to have better ODS knowledge than those with primigravida. Also, higher gravidity was reported a significant predictor in some previous studies. ^27^^,^^33^

Our study also found that pregnant women who had medical personnel as a source of ODS knowledge tended to have better ODS knowledge than those who obtained information from women’s friends or other media. Medical personnel are a good source of information for expecting mothers. This could be partially reflected by a lower gestational age at the first antenatal care (ANC) visit and the higher number of ANC visits since these women had more time spent in the clinic, and thus had more opportunities to obtain important information from medical personnel. Although our study cannot demonstrate that these two variables were predictors of better ODS knowledge, many other studies showed the association, e.g., studies in Thailand, ^10^ Congo, ^33^ Saudi Arabia, ^41^ and Ethiopia. ^16^^–^^20^^,^^23^^,^^38^

The strengths of our study include a large sample size and only one well-trained research assistant collecting the data to minimize the inter-observer variation. Furthermore, data collection setting and process were similar for all participants and were considerably optimal since it took place in a private room during the waiting time in the antenatal care clinic. In addition, our study design was similar to other studies in the same region, allowing comparison of data between countries with similar context.

Nevertheless, our study has some limitations. The study was cross-sectional, so we cannot establish a causal relationship between variables. We did not evaluate some variables, i.e., the number of family member and the region of residence, which were reported as significant predictors in previous studies. ^18^^,^^31^ Some variables might also affect the knowledge, such as interval between pregnancy, birth preparedness, accessibility to healthcare services, and medical expense subsidies. Further study focusing on the relationship between pregnant woman’s knowledge and her intimate partner’s knowledge should be conducted.

## Conclusion

Our study demonstrates the fair prevalence of mothers with good knowledge of ODS in Thailand’s rural areas. We found that participants’ age of at least 20 years, higher education, and reporting medical personnel as the source of ODS knowledge were predictors of good ODS knowledge. Identifying those at risk for fair and poor ODS knowledge and prompt management for the vulnerable subgroups might help decrease maternal mortality in this region. Further research and educational program are needed to raise the knowledge of ODS, aiming to reduce maternal mortality.

## Data Availability

Harvard Dataverse: Knowledge of obstetric danger signs and associated factors among pregnant women attending antenatal care services at Thai community hospital datasetEN version, https://doi.org/10.7910/DVN/OEFYS6. ^42^ This project contains the following underlying data:
• Knowledge of obstetric danger signs and associated factors among pregnant women attending antenatal care services at Thai community hospital datasetEN version.tab Data are available under the terms of the Creative Commons Zero “No rights reserved” data waiver (CC0 1.0 Public domain dedication).
