# Supplementary material for: Knowledge of obstetric danger signs and associated factors among pregnant women attending antenatal care services at Thai community hospital
Source: F1000Res. 2023 Oct 27;12:851. Originally published 2023 Jul 19. [Version 2] doi: 10.12688/f1000research.131267.2 (PMC10643879; doi:10.12688/f1000research.131267.2)
Supplement: Peer review history

# Reviewer report: Reviewer response for version 2

**DOI:** 10.5256/f1000research.157811.r218766

**Published:** 2023-11-13

**Authors:** Natnita Mattawanon

**Affiliations:** 1 Department of Obstetrics and Gynecology, Faculty of Medicine, Chiang Mai University, Chiang Mai, Chiang Mai, Thailand

I do not have any further questions.

Is the work clearly and accurately presented and does it cite the current literature?

Yes

If applicable, is the statistical analysis and its interpretation appropriate?

Yes

Are all the source data underlying the results available to ensure full reproducibility?

Yes

Is the study design appropriate and is the work technically sound?

Yes

Are the conclusions drawn adequately supported by the results?

Yes

Are sufficient details of methods and analysis provided to allow replication by others?

Yes

Reviewer Expertise:

Clinical OB&GYN

I confirm that I have read this submission and believe that I have an appropriate level of expertise to confirm that it is of an acceptable scientific standard.

# Reviewer report: Reviewer response for version 2

**DOI:** 10.5256/f1000research.157811.r218765

**Published:** 2023-11-01

**Authors:** Jerome K Kabakyenga

**Affiliations:** 1 Maternal Newborn and Child Health Institute, Mbarara University of Science and Technology, Mbarara, Western Region, Uganda

The authors have attended to the issues I raised earlier.

 I confirm my approval.

Is the work clearly and accurately presented and does it cite the current literature?

Yes

If applicable, is the statistical analysis and its interpretation appropriate?

Yes

Are all the source data underlying the results available to ensure full reproducibility?

Yes

Is the study design appropriate and is the work technically sound?

Yes

Are the conclusions drawn adequately supported by the results?

Yes

Are sufficient details of methods and analysis provided to allow replication by others?

Yes

Reviewer Expertise:

Reproductive Maternal Newborn Child Adolescent Health

I confirm that I have read this submission and believe that I have an appropriate level of expertise to confirm that it is of an acceptable scientific standard.

# Reviewer report: Reviewer response for version 1

**DOI:** 10.5256/f1000research.144090.r188939

**Published:** 2023-10-17

**Authors:** Natnita Mattawanon

**Affiliations:** 1 Department of Obstetrics and Gynecology, Faculty of Medicine, Chiang Mai University, Chiang Mai, Chiang Mai, Thailand

This study presented data concerning the extent of awareness among pregnant individuals in a rural area of Thailand regarding indicators of potential pregnancy-associated risks, and whether they possess acquaintance with this particular information. 

 The investigators employed both questionnaire surveys and interview methodologies to acquire the dataset. 

 The result revealed that approximately 10% of the participants exhibited a deficient knowledge of Obstetric Danger Signs (ODS), while an additional 25% attained a moderate level of understanding. Subpopulations characterized by an age below 20 years, limited educational attainment, and reliance on familial or peer networks as their primary information sources demonstrated significantly diminished levels of knowledge. Furthermore, this investigation highlighted the frequent oversight of critical indicators of preeclampsia, such as visual disturbances, epigastric discomfort, and convulsions, by the participants. This underscores the scope for healthcare professionals and governmental bodies to implement precise interventions to improve this situation. 

 From my perspective, this paper offers enhanced insights into the landscape of pregnancy care within rural regions of developing countries. Moreover, it highlights a targeted area for potential development. 

 There is, however, one aspect requiring further explanation within the Methodology section. 

 1. what is the rationale behind establishing the threshold for "good knowledge" at 75%? 

 The derivation of this specific percentage warrants further clarification.

Is the work clearly and accurately presented and does it cite the current literature?

Yes

If applicable, is the statistical analysis and its interpretation appropriate?

Yes

Are all the source data underlying the results available to ensure full reproducibility?

Yes

Is the study design appropriate and is the work technically sound?

Yes

Are the conclusions drawn adequately supported by the results?

Yes

Are sufficient details of methods and analysis provided to allow replication by others?

Yes

Reviewer Expertise:

Clinical OB&GYN

I confirm that I have read this submission and believe that I have an appropriate level of expertise to confirm that it is of an acceptable scientific standard, however I have significant reservations, as outlined above.

# Reviewer report: Reviewer response for version 1

**DOI:** 10.5256/f1000research.144090.r194222

**Published:** 2023-10-17

**Authors:** Jerome K Kabakyenga

**Affiliations:** 1 Maternal Newborn and Child Health Institute, Mbarara University of Science and Technology, Mbarara, Western Region, Uganda

Title: Knowledge of obstetric danger signs and associated factors among pregnant women attending antenatal care services at Thai community hospital. The aim of the study was to identify (determine) the prevalence of good ODS knowledge and associated factors among pregnant women attending antenatal services at a Thai community hospital.

 Abstract:

 Well structured and is a summary of the body of the manuscript.

 Key words are missing - obstetric danger signs

 Introduction:

 paragraph -2 the statement.."differs among countries, ranging from 23.8 deaths per  100,000 live births in the US to 442 deaths per 100,000 births in Africa". The authors chose to give a MMR lowest starting with USA - why not start with the country (ies) with the lowest MMR (<5/100,000) e.g. from a publication "Trends in maternal mortality 2000 to 2020: estimates by WHO, UNICEF, UNFPA, World Bank Group and UNDESA/Population Division".

 Paragraph 4 - there are several statements in this paragraph that need to be supported with references (sentences 2,3,4,5).

 Results:

 It would be more informative if Table 1 is about the sociodemographic, obstetric data of participants.

 Discussion:

 Is based on results of the study.

 Conclusion:

 Is derived from the results and discussion of the study.

 General:

 There is need to improve on the grammatical layout of the manuscript.

Is the work clearly and accurately presented and does it cite the current literature?

Yes

If applicable, is the statistical analysis and its interpretation appropriate?

Yes

Are all the source data underlying the results available to ensure full reproducibility?

Yes

Is the study design appropriate and is the work technically sound?

Yes

Are the conclusions drawn adequately supported by the results?

Yes

Are sufficient details of methods and analysis provided to allow replication by others?

Yes

Reviewer Expertise:

Reproductive Maternal Newborn Child Adolescent Health

I confirm that I have read this submission and believe that I have an appropriate level of expertise to confirm that it is of an acceptable scientific standard.
